# Complete Genome Sequence of Bacillus safensis Strain 3A, a Heavy Metal-Resistant Bacterium Isolated from Contaminated Estuarine Sediment in Brazil

**DOI:** 10.1128/MRA.00268-21

**Published:** 2021-04-22

**Authors:** Thaiane Defalco, Ana L. S. Vasconcelos, Armando C. F. Dias, Leticia Barrientos, Ângelo F. Bernardino, Fernando Dini Andreote, Kattia Núñez-Montero

**Affiliations:** a Escola Superior de Agricultura Luiz de Queiroz, Universidade de São Paulo, São Paulo, Brazil; b Laboratorio de Biologia Molecular Aplicada, Centro de Excelencia en Medicina Traslacional, Universidad de La Frontera, Temuco, Chile; c Universidade Federal do Espírito Santo, Vitória, Brazil; d Centro de Investigación en Biotecnologia, Instituto Tecnológico de Costa Rica, Cartago, Costa Rica; Queens College CUNY

## Abstract

Bacillus safensis 3A was isolated from a contaminated estuarine sediment sample with mine tailing from the Samarco dam disaster, which occurred in 2015 in Minas Gerais State, Brazil. We report here a draft genome sequence (3.6 Mb) of this bacterial strain. *B. safensis* exhibited strong resistance to heavy metals.

## ANNOUNCEMENT

Mining industry activity is associated with heavy metal discharge into the environment. In 2015, the rupture of the mining tailings dam in Mariana, Minas Gerais, southeastern Brazil, released about 50 million m^3^ of toxic sludge (containing iron and manganese) into the river Rio Doce (1). The mud traveled for more than 650 km, causing severe damage in protected areas (2–4), and the incident was thus considered the most devastating environmental disaster in Brazilian history.

Bacillus safensis 3A was isolated from the toxic sludge of the estuarine sediments in a tryptic soy agar (TSA) culture containing 1 ml/liter of nystatin and Mn (3,200 μg/ml). This species is a Gram-positive, spore-forming, aerobic, and chemoheterotrophic bacterium that can colonize extreme environments due to its high tolerance to salts and heavy metals (5). For whole-genome sequencing, genomic DNA from *B. safensi*s 3A was obtained using the microbial UltraClean extraction kit (Mo Bio Laboratories, USA) from a colony grown overnight in a TSA plate at 30°C. Genomic libraries were prepared with the rapid barcoding sequencing SQK-RBK004 kit (Oxford Nanopore Technologies [ONT], UK) for sequencing on a minION platform using the MinKNOW software, followed by base calling and conversion of the raw data to FASTQ format with Guppy v.3.6.0 (https://staff.aist.go.jp/yutaka.ueno/guppy/). The recovered data resulted in 64,562 ONT reads with a read length *N*_50_ value of 4,319 bp. A Nextera XT library prep kit (Illumina, Inc., CA) was used for Illumina sequencing in a MiSeq X v.3 platform with the paired-end method, using a 350-bp average size in 2 × 150-bp sequencing, resulting in a total of 356,168 Illumina reads. Low-quality ONT reads and adapters (Phred score, <10; length, <5,000 bp) were trimmed with Porechop v.0.2.4 and NanoFilt v.2.8.0 (6), respectively. The Illumina reads were filtered with fastp v.0.20.1 (7) with default parameters. A hybrid *de novo* genome assembly was performed using Unicycler v.0.4.8, which includes the removal of overlapping sequences and genome polishing in its pipeline with the SPAdes optimizer and Pilon, respectively (8). The assembly quality was evaluated with Quast v.5.0.2 (9) and CheckM v1.1.3 (10). Annotation was performed with the NCBI Prokaryotic Genome Annotation Pipeline (11).

The genome assembly resulted in 2 contigs, a unique chromosome of 3,721,772 bp (not rotated), and a plasmid of 1,999 bp. This genome showed 61× coverage, 41.7% GC content, and high quality, having 99.59% completeness and ≤5% contamination (0.21%). The 3A strain was identified as a Bacillus safensis species by 16S rRNA BLASTn comparison (12) and average nucleotide identity (ANI) analysis in the JSpeciesWS online server (13), which showed 98.46% ANI blast (ANIb) and 98.57% ANI analysis using MUMmer3 (ANIm) identity with *B. safensis* PgKB20. The annotation showed that the 3A genome has 3,835 coding sequences and 105 RNAs, including 53 tRNAs and 8 rRNAs. Among the annotated genes, multiple putative genes of metal and antibiotic resistance were identified, i.e., cobalt-zinc-cadmium resistance protein CzcD, copper resistance proteins CopD, CopC, and Bcr/CflA, and a β-lactamase antibiotic resistance gene (in the plasmid).

### Data availability.

This project has been deposited at DDBJ/ENA/GenBank under the following accession numbers: PRJNA667924 (BioProject), SAMN17221404 (BioSample), CP067376 (genome), MW647491.1 (plasmid), and SRX10206807 and SRX10206806 (raw sequencing data).
